# Clinical evaluation of AI-assisted screening for diabetic retinopathy in rural areas of midwest China

**DOI:** 10.1371/journal.pone.0275983

**Published:** 2022-10-13

**Authors:** Shaofeng Hao, Changyan Liu, Na Li, Yanrong Wu, Dongdong Li, Qingyue Gao, Ziyou Yuan, Guanyan Li, Huilin Li, Jianzhou Yang, Shengfu Fan

**Affiliations:** 1 Department of Ophthalmology, Heji Hospital Affiliated with Changzhi Medical College, Changzhi, China; 2 Postgraduate Department, Changzhi Medical College, Changzhi, China; 3 Department of Ophthalmology, Affiliated Hospital of Jiangxi University of Chinese Medicine, Nanchang, China; 4 Department of Ophthalmology, No. 1 People’s Hospital of Huaihua, Huaihua, China; 5 Department of Public Health and Preventive Medicine, Changzhi Medical College, Changzhi, China; 6 Department of Foreign Languages, Changzhi Medical College, Changzhi, China; University of Warmia, POLAND

## Abstract

**Background:**

Although numerous studies have described the application of artificial intelligence (AI) in diabetic retinopathy (DR) screening among diabetic populations, studies among populations in rural areas are rare. The purpose of this study was to evaluate the application value of an AI-based diagnostic system for DR screening in rural areas of midwest China.

**Methods:**

In this diagnostic accuracy study, diabetes mellitus (DM) patients in the National Basic Public Health Information Systems of Licheng County and Lucheng County of Changzhi city from July to December 2020 were selected as the target population. A total of 7824 eyes of 3933 DM patients were enrolled in this screening; the patients included 1395 males and 2401 females, with an average age of 19–87 years (63±8.735 years). All fundus photographs were collected by a professional ophthalmologist under natural pupil conditions in a darkroom using the Zhiyuan Huitu fundus image AI analysis software EyeWisdom. The AI-based diagnostic system and ophthalmologists were tasked with diagnosing the photos independently, and the consistency rate, sensitivity and specificity of the two methods in diagnosing DR were calculated and compared.

**Results:**

The prevalence rates of DR according to the ophthalmologist and AI diagnoses were 22.7% and 22.5%, respectively; the consistency rate was 81.6%. The sensitivity and specificity of the AI system relative to the ophthalmologists’ grades were 81.2% (95% confidence interval [CI]: 80.3% 82.1%) and 94.3% (95% CI: 93.7% 94.8%), respectively. There was no significant difference in diagnostic outcomes between the methods (χ2 = 0.329, *P* = 0.566, *P*>0.05), and the AI-based diagnostic system had high consistency with the ophthalmologists’ diagnostic results (κ = 0.752).

**Conclusion:**

Our research demonstrated that DR patients in rural area hospitals can be screened feasibly. Compared with that of the ophthalmologists, however, the accuracy of the AI system must be improved. The results of this study might lend support to the large-scale application of AI in DR screening among different populations.

## Introduction

Diabetic retinopathy (DR) is a complication of progressive diabetic mellitus (DM), capable of damaging retinal vessels, resulting in microaneurysms, retinal exudation, vitreous hemorrhage, retinal traction and so on, and eventually leading to vision loss and even blindness in DM patients. The prevalence of DR among DM patients is 34.6% worldwide, 10.2% of whom suffer visually impaired retinopathy [1]. In China, the prevalence of DR is 23%; among individuals with DR, nonproliferative DR accounts for 19.1%, and the vision-threatening proliferative type accounts for 2.8% [2]. In terms of age of onset, DR peaks between 60 years and 69 years; it also increases with the course of the primary disease [3]. It is estimated that approximately 40%-45% of DM patients can be detected and diagnosed in time; that is, only half of them will learn that they have DR [4]. Additionally, the onset of DR is insidious, with few apparent symptoms. For most DM patients who come to the hospital for fundus lesion examination, DR has already progressed to a moderate or even severe stage [5]. Thus, it is of great importance for DM patients to receive appropriate primary health care and early systematic screening in the community to prevent vision loss [6].

To date, many methods for DR screening have been proposed, including traditional screening methods, telemedicine and artificial intelligence (AI). Traditional screening methods include fundus photography, scanning laser ophthalmoscopy (SLO), fundus fluorescein angiography (FFA), and optical coherence tomography (OCT). However, neither traditional screening methods nor telemedicine can meet the needs of a growing number of DM patients because ophthalmologists are required to analyze and classify their fundus photos; the accuracy of this classification is affected by the ophthalmologist’s clinical experience, subjectivity, and fatigue, and the entire process is time consuming and requires a large amount of engineering [7].

AI is a relatively new branch of computer science used to research and develop technologies and application systems based on human intelligence. The rapid development of computers has led to the gradual incorporation of AI as the mainstream technology in various scientific research fields [8]. AI was first introduced into the medical field in the 1970s; since then, a number of AI systems have been developed. Improvements in hardware computing power, the continuous accumulation of data and the proposal and development of deep learning (DL) theory has resulted in major breakthroughs in intelligent medicine through AI technology [9]. Specifically, AI has allowed great progress in ophthalmology. Today, a variety of DR intelligent diagnosis techniques, usually based on machine learning (ML) technology, are implemented, mainly realized by DL technology. ML describes algorithms that become more accurate in predicting results in an application without explicit programming [10]. AI diagnosis systems for detecting and classifying DR learn from thousands of retina images of different levels of DR from the system. After recognizing a large number of images marked with pathological changes, the machine learns to grade DR [11]. AI can be used to grade retinal images taken by traditional fundus cameras and to determine which DR patients need to be referred to ophthalmologists in a timely manner [12].

A large number of studies [9, 13, 14] have shown that AI diagnosis systems have high sensitivity and specificity. Therefore, they can detect and diagnose DR in a timely manner, reducing the workload of ophthalmologists, relieving the burden of a lack of ophthalmologists, and improving the efficiency of DR screening worldwide, thereby reducing the burdens on society, medical systems and patients. Scholars have also made meaningful explorations of AI diagnosis systems in China [5, 15, 16]. However, most of these studies were performed in community hospitals in large cities. In China, the prevalence of DM in rural areas is higher than that in urban areas and may be affected by economic conditions, uneven distribution of medical resources and education level [3]. Most DM patients cannot afford the lifelong follow-up and treatment of DR; 87% of them go to medical institutions below the county level, and 70% of them do not receive standard fundus examinations and treatments. Early follow-up and treatment are a great challenge for DR patients in rural areas, where necessary ophthalmic equipment is lacking [15]. Due to a shortage of professional ophthalmologists, poor awareness of medical treatment, and the fact that this medical condition is relatively rare in rural areas, the eye conditions of individuals in rural areas might be more severe at the time of detection compared with those of their counterparts in urban areas. Therefore, the conduction of large-scale DR screening in these populations is urgent. The development of AI technology may provide a promising means for the early diagnosis and treatment of DM among rural populations. Searches in the literature, however, have revealed that there is currently a lack of large-scale screening in rural areas, and therefore, how to apply AI technology for rural populations remains to be explored.

To solve these problems, we conducted AI diagnosis system-assisted DR screening in rural areas of Changzhi city, Central and Western China, to evaluate the accuracy of using AI in DR screening in rural areas. This study may be of great application value not only for other rural areas of midwest China but also for the regions of other countries with similar socioeconomic backgrounds.

## Materials and methods

### Study design

**Fig 1:**
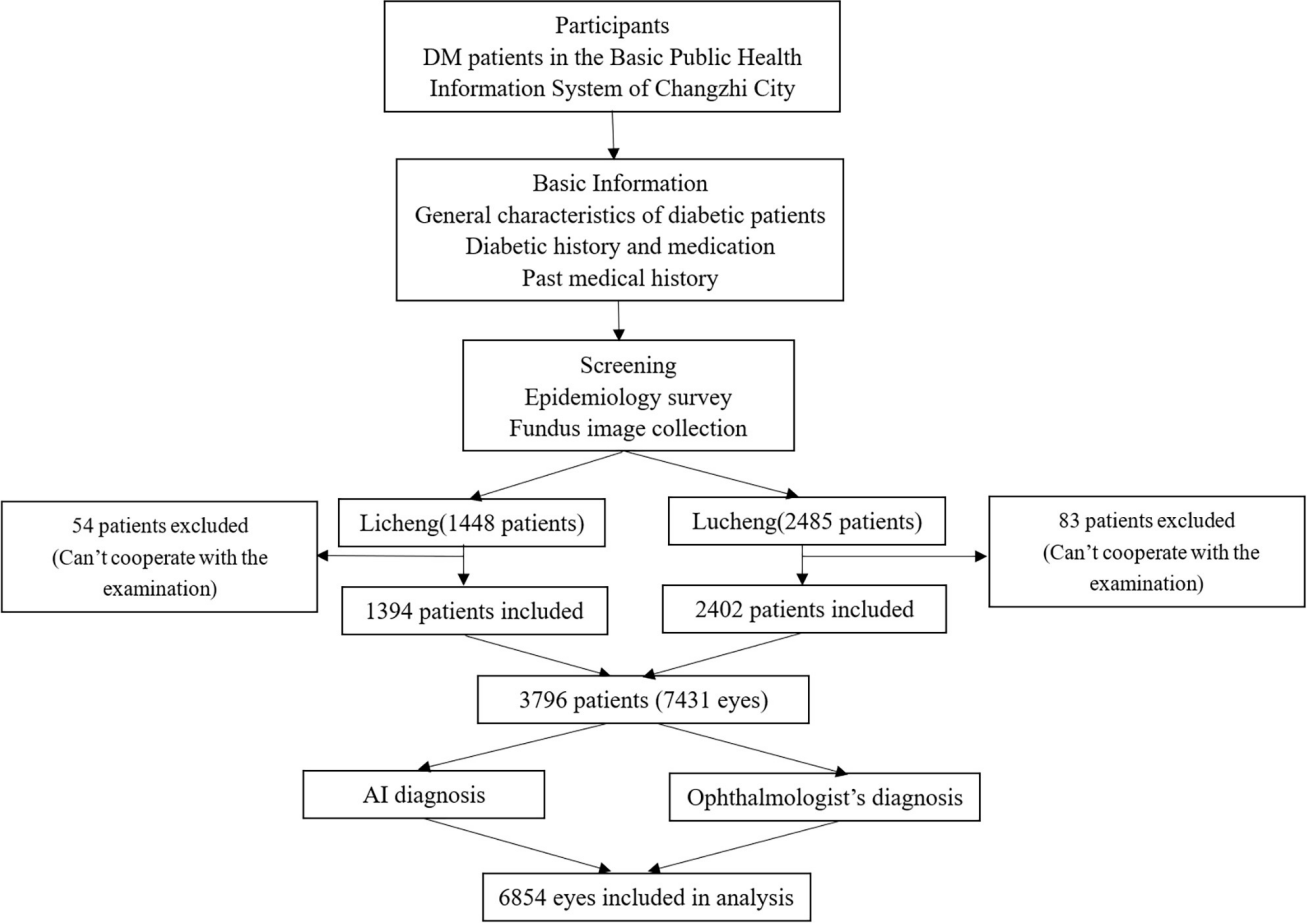
Flow chart of the current study.

This study is a prospective clinical trial of DR screening based on an AI diagnostic system (clinical registration no., ChiCTR200003283). An epidemiological investigation was conducted with all diabetic patients who met the inclusion criteria. Fundus images were collected and uploaded to the AI system, and the results were automatically graded according to the international DR staging system. The fundus images were collected and graded by an ophthalmologist, and the results were compared with those of the AI system to evaluate the reliability of AI. The flow chart of this study is shown in Fig 1.

### Ethical statement

This study was approved by the Evaluation Committee of Changzhi Heji Hospital in Shanxi Province (approval no., 2019016) (Chinese Clinical Trial Registry, ChiCTR200003283; http://www.chictr.org.cn/showproj.aspx?proj=53446, registered on May 13, 2020). The procedures of this study were performed in accordance with the Declaration of Helsinki. All participants signed informed consent forms.

### Research object

Changzhi city contains 11 counties. Among them, the populations of Lucheng and Licheng are approximately 219,000 and 134,200, respectively, and the proportions of people living below the county level are approximately 40% and 54%, respectively. According to the *Statistical Yearbook 2021* released by the Shanxi Provincial Bureau of Statistics, among the 117 counties in Shanxi Province in 2020, Lucheng and Licheng ranked 46th and 107th, respectively, with a per capita GDP of 58,600 yuan and 25,100 yuan, respectively; thus, Lucheng and Licheng represent counties at a relatively high and low economic level, respectively. Studies have shown that there is an independent correlation between the incidence of DR and personal economic status [17]. In addition, most of the territories of Licheng and Lucheng are hills and mountains; due to the complex topography and low resident density, typical of rural villages in China, the screening group comprised DM patients from the two counties. DM patients who had already been registered in the National Basic Public Health Information System of nonurban residents below the county level in Licheng and Lucheng of Changzhi city from July 1, 2021, to November 30, 2021, were included as the target population. A total of 3933 patients participated in this screening, 137 of whom were excluded because they could not complete the examinations due to the presence of serious physical and/or other conditions; 3796 DM patients were included, 1295 males (34.1%) and 2501 females (65.9%), with an average age of 19–87 years (63.03±8.718 years). For these patients, 3722 right eyes and 3709 left eyes were included, for a total of 7431 eyes. Among them, 577 eyes were reported as "unreadable" by the system AI due to severe cataracts and corneal leukoplakia; ultimately, 6854 eyes were included in this study.

Inclusion criteria: (1) registration in the National Basic Public Health Information System, awareness of the purpose of the clinical trial and voluntary participation, and signing of the informed consent form (for elderly patients, consent may be obtained from their guardians); and (2) type 1 or type 2 DM, male or female sex, and age ≥ 18 years.

Exclusion criteria: (1) inability to cooperate with the examination due to serious physical and/or other diseases; (2) refractive media opacity (such as corneal ulcers, corneal leukoplakia, severe cataracts, vitreous hemorrhage and massive exudation, according to fundus examination); (3) poor quality of the acquired image; (4) incomplete screening data, lack of AI results or physician results; and (5) inability to determine DR grading after fundus laser surgery.

## Methods

### Examination methods

**Fig 2:**
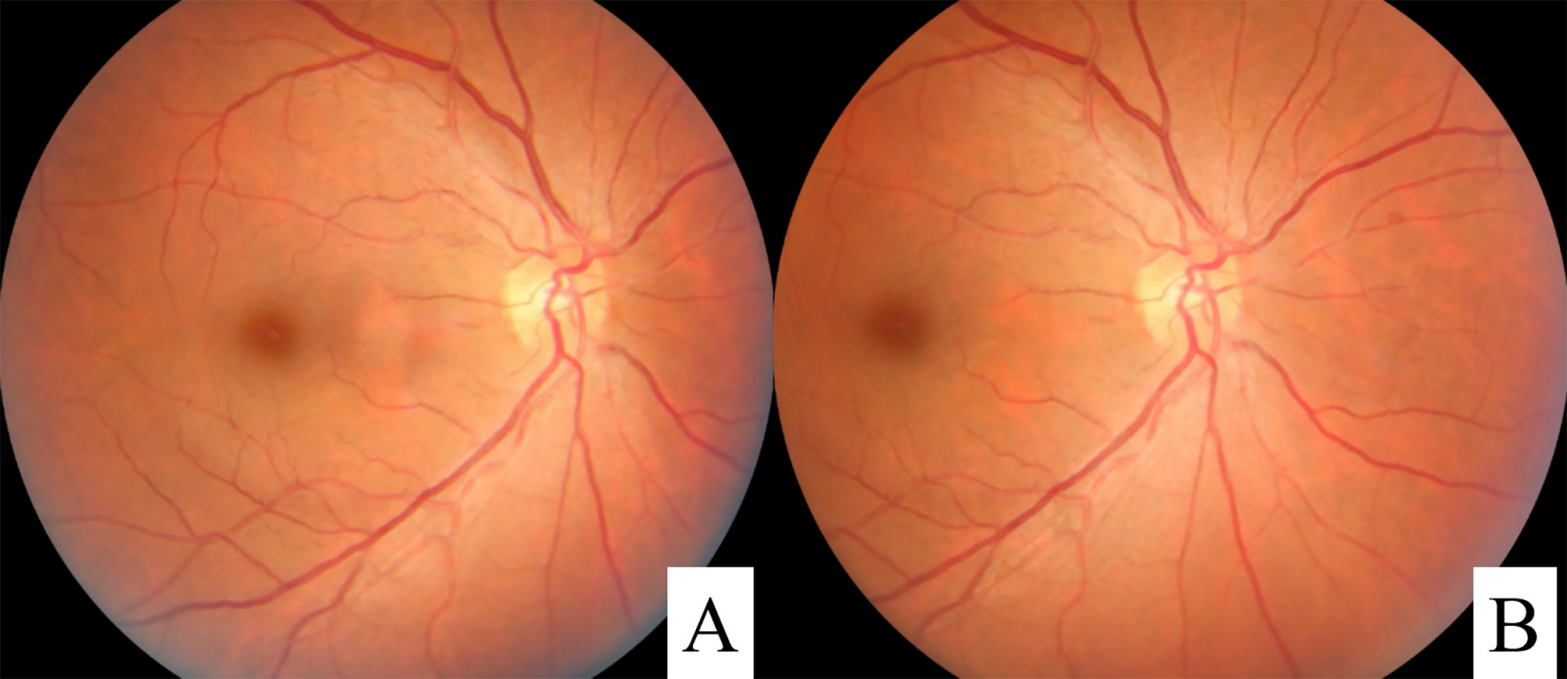
Examples of fundus images. (A): Fundus image centered on the macula; (B) Fundus image centered on the optic disc.

This prospective study was planned to begin on July 1, 2021, and end on November 30, 2021. The contact information of all the included patients was obtained through the Basic Public Health Information System of Changzhi city. All primary care physicians in the study area are required to register cases of DM in this system. The DM patients were contacted by the general practitioner who is in charge of the community (village) where the patients live. If they could not be contacted, door-to-door notifications were made to each patient through the village committee. DR screening was performed at the local community hospital at an appointed time. The same Zeiss VISUCA500 fundus camera (Carl Zeiss, Jena, Germany) was used for both counties. Preliminary data, such as age, sex, DM medical history and medication method, and whether DR was diagnosed, were collected. The primary examination was completed with a slit lamp by the chief physician, and patients with corneal leukoplakia, severe cataracts and other eye diseases were excluded. For each eye of every patient, two photographs, including one 45-degree photo centered on the macula (Fig 2A) and the other on the optic disc (Fig 2B), were taken by a senior professional optometrist (at least 10 years’ working experience) under natural pupil conditions in a darkroom. If there were obvious abnormalities in the photo, peripheral fundus images were taken. Then, these fundus images were used as the photo library for screening and uploaded into the AI system for DR-based screening and grading. Images of the same eye were evaluated by two attending physicians with more than 10 years of experience. Each ophthalmologist was required to read the images independently; if the results were consistent, they were used for DR grading; if the results were inconsistent, a more experienced chief physician was consulted and made the final diagnosis. The final DR grading was performed using the more severe eye of each patient, and the consistency between the AI system and the physicians was determined, with the physicians’ reading outcomes considered as the gold standard.

### DR grading and lesion labeling

The less severely diseased eye of each patient was used for the final diagnosis following a DR grading standard based on the International Clinical Diabetic Retinopathy Scale (ICDRs) [18]. The fundus conditions of the DM patients were divided into 5 stages: no apparent DR (no abnormalities), mild nonproliferative diabetic retinopathy (NPDR, microaneurysms only), moderate NPDR (more than only microaneurysms but less than severe nonproliferative DR), severe NPDR (any of the following: intraretinal hemorrhages (≥20 in each quadrant); definite venous beading (in two quadrants); intraretinal microvascular abnormalities (in 1 quadrant); no signs of proliferative retinopathy) and proliferative diabetic retinopathy (PDR, severe nonproliferative DR with any or both of the following: neovascularization and vitreous/preretinal hemorrhage). The marked lesions included microaneurysms, cotton wool spots, hard exudate, soft exudate, intraretinal hemorrhage, venous beading, fibrous proliferative membrane, retinal detachment, vitreous hemorrhage and other fundus abnormalities. For patients who had been treated with laser surgery, the AI-based diagnostic system can distinguish whether the eye is stable or unstable and develop a follow-up plan. The AI-based diagnostic system can also report fundus diseases other than DR, such as high myopia and age-related macular degeneration. As a single fundus image cannot be used to confirm the clinical significance of macular cystoid edema, its clinical grade and lesion labeling were not included in this study.

### Intelligent auxiliary diagnosis and lesion labeling

**Fig 3:**
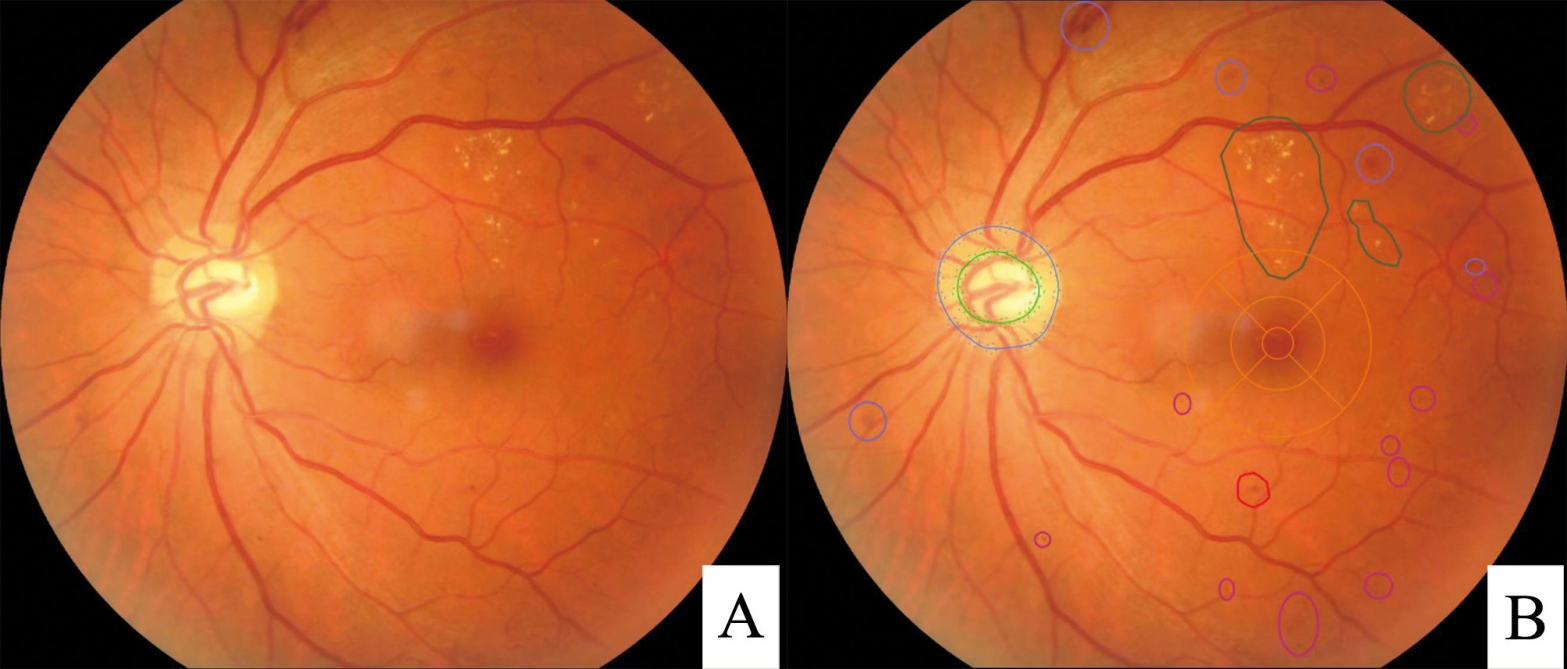
Working interface and lesion labeling of the Zhiyuan Huitu image AI analysis software (EyeWisdom). (A): Zhiyuan Huitu image AI analysis software (EyeWisdom) interface; (B): In the fundus image, the green range indicates hard exudate, the purple range indicates retinal hemorrhage, the pink range indicates a microaneurysm, and the red range indicates suspected bleeding or microaneurysm.

The Zhiyuan Huitu fundus image AI analysis software EyeWisdom (Visionary Intelligence Ltd., Beijing, China, certified by the National Medical Products Administration, certification number: 20213210422, https://www.nmpa.gov.cn/directory/web/nmpa/xxgk/ggtg/ylqxpzhzhcchpgg/20210714171346191.html) used in this study is an AI-based fundus imaging analysis software integrated into the picture archiving and communication system (PACS), an online retinal image analysis system, that can upload retinal photos, automatically grade DR, and produce single page reports. It has been tested with many different types of fundus cameras during the development of the model, such as the Topcon TRC-NW400, Raymond TNF506, and Center Vue DRS, and has shown good results, with both sensitivity and specificity reaching more than 93%, showing high homogeneity. The ophthalmologist who took the fundus images collected and uploaded patient information to the PACS, including patient name, sex, age, time of diagnosis of DM, visual acuity, and intraocular pressure, and matched it with each image [19]. The condition of the eye under slit lamp examination were recorded. The EyeWisdom system was developed using a database of 25,297 fundus images (21,512 from the Kaggle database and 3785 from Henan Eye Hospital and Peking Union Medical College Hospital), and more than 1 million retinopathy cases were manually marked by professional fundus specialists and specifically used to identify DR. Deep learning and network data training were realized through the YOLO detection system [20]. The AI software uses the Inception-V3 convolutional neural network (CNN) to grade DR automatically. The Inception-V3 network contains branch structures with different-sized convolution kernels, which can extract branch structures with different sizes and lesion features so that it can better extract lesion features in DR grading. Fig 3 shows the working interface of the AI analysis software for fundus imaging and lesion labeling of Zhiyuan Hui maps.

### Verification of AI screening results

The ophthalmologists’ reading outcomes were used as the gold standard for evaluating the effectiveness of AI screening. Specifically, fundus photos of the same eye were evaluated by two attending physicians with more than 10 years of experience. Each ophthalmologist was required to read the photos independently, and the results were compared. When the two physicians’ diagnosis results were consistent, the diagnosis results were used as the grading of DR. If the results of the two attending physicians were inconsistent, a more experienced chief physician was consulted and made the final diagnosis. The consistency between the AI diagnosis system and the ophthalmologists’ diagnosis results was compared.

### Statistical analysis

SPSS 22.0 statistical software was used to analyze the data. Categorical data are presented as the number (percentage), and the χ2 test was performed to compare between groups. Continuous data are presented as the mean ± deviation, and the *t* test was used to compare between groups. *P*<0.05 was considered to be significantly different.

The performance of the AI algorithm was evaluated using the ophthalmologist’s diagnostic results as a reference standard. Kappa (κ) statistics were used to quantify and evaluate the degree of consistency between the AI-based diagnostic system and the ophthalmologist’s diagnosis results: κ≥0.75 indicated good consistency between the two methods; κ between 0.40–0.75 indicated that the consistency of the diagnostic results of the two methods was moderate; and κ<0.4 indicated poor consistency in the diagnostic results between the two methods. Sensitivity and specificity were used to evaluate the accuracy of the AI-based diagnostic system in screening DR.

## Results

### General data

A total of 3933 patients were initially involved in DR screening in this study, and 3796 DM patients were finally included; 137 patients were excluded because they could not complete the examinations due to the presence of serious physical and/or other diseases. The included patients consisted of 1394 patients from Licheng and 2402 patients from Lucheng, aged from 19 to 87 years, with an average age of 63.03±8.718 years. Among them, 1395 were male, including 519 from Licheng and 876 from Lucheng, and 2401 DM patients were female, including 875 from Licheng and 1526 from Lucheng. A total of 3722 right eyes and 3709 left eyes were initially enrolled, for a total of 7431 eyes. Of these, 577 (7.76%) eyes were reported as "unreadable" by the AI software, including 481 eyes reported as "unreadable" by the ophthalmologists due to severe cataracts and/or corneal leukoplakia or other reasons that led to refractive media opacity (S1 File). A total of 6854 eyes were finally included in the study.

There were no significant differences in age (*t* = 1.425, *P* = 0.075; Table 1; S1 File) or sex (χ^2^ = 0.220, *P* = 0.639; Table 1) between the two counties. No significant difference was observed in the prevalence of DR (Licheng vs. Lucheng: 35.85% vs. 38.71%; χ^2^ = 3.051, *P* = 0.081; Table 1) according to the ophthalmologists’ diagnosis, which was used as the gold standard.

**Table 1:** General data and prevalence of DR in the two counties.

	Licheng	Lucheng	*P* value
Sex			χ^2^ = 0.220, *P* = 0.639
Male (n)	519	876	
Female (n)	875	1526	
Age (years)	63.36±8.696	62.46±8.729	*t* = 3.065, *P* = 0.732
DR prevalence (n/%)	500/35.85%	930/38.71%	χ^2^ = 3.051, *P* = 0.081

Note: DR: diabetic retinopathy.

### Consistency between the AI and the physician diagnostic groups

**Fig 4:**
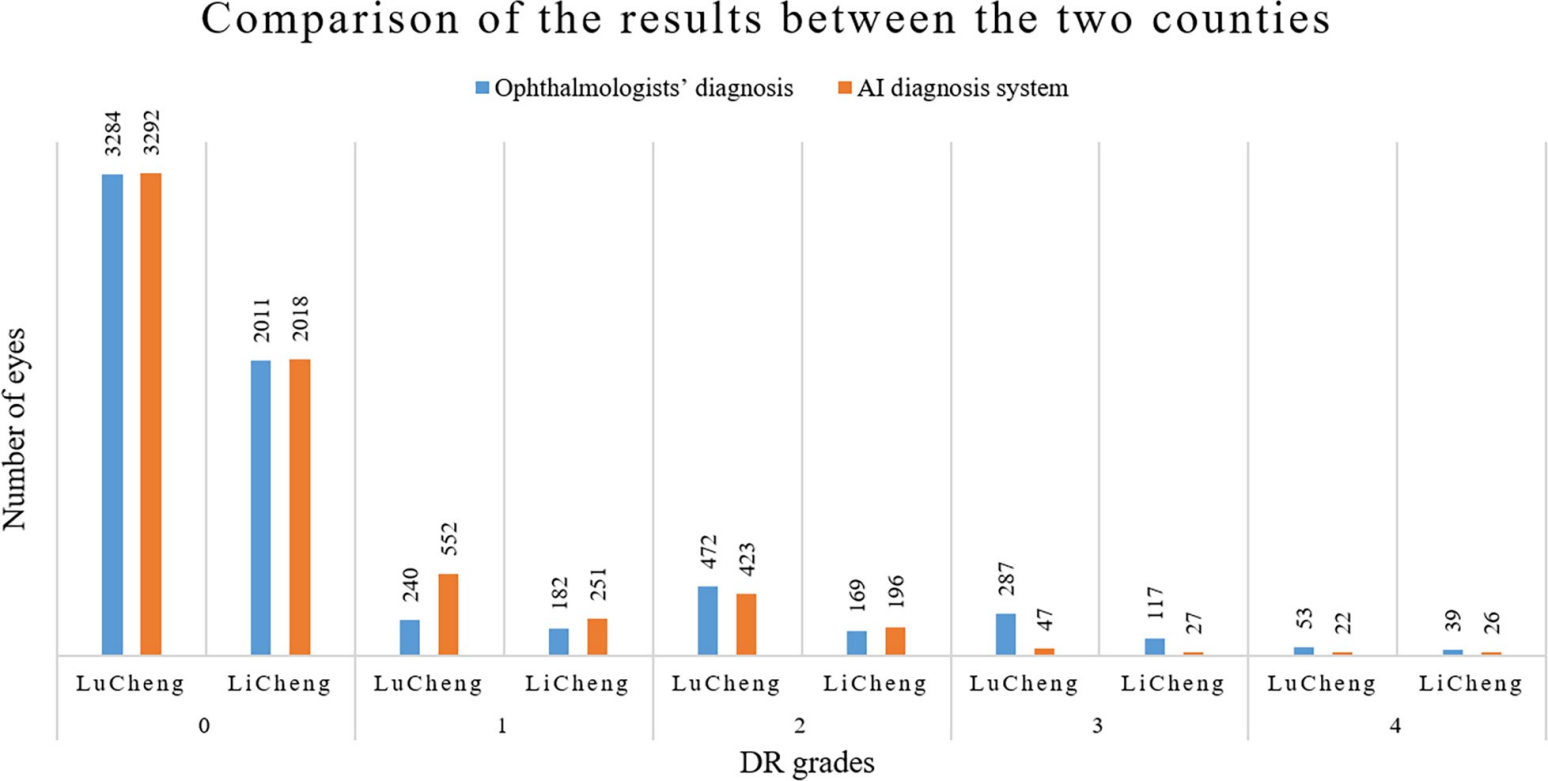
Comparison of the results between the two counties.

Among 6854 eyes, the ophthalmologists diagnosed no DR (NDR) in 5295 (77.3%); the remaining 1559 eyes had different degrees of DR (22.7%). The AI diagnosed NDR in 5310 eyes (77.4%); the remaining 1544 eyes (22.5%) had different degrees of DR. Additionally, to provide a more convincing result, we also analyzed the 577 eyes excluded by the AI system. The detailed results are summarized in Table 2, and Fig 4 shows a comparison of the results between Lucheng and Licheng. The consistency rate, hereafter referring to the percentage of cases diagnosed consistently between the AI and the ophthalmologists, was 81.6%, meeting the requirements for primary screening.

**Table 2:** Comparison of the diagnoses between the ophthalmologists and AI according to the International Stages of Diabetic Retinopathy Scale.

Ophthalmologists’ diagnosis	AI diagnosis system						Total
	No DR	Mild NPDR	Moderate NPDR	Severe NPDR	PDR	U	
No DR	5005	237	53	0	0	65	5360
Mild NPDR	99	265	53	2	3	25	447
Moderate NPDR	151	235	247	3	5	6	647
Severe NPDR	47	59	242	47	9	0	404
PDR	8	7	24	22	31	0	92
U	0	0	0	0	0	481	481
Total	5310	803	619	74	48	577	7431

Note: DR: diabetic retinopathy, NPDR: nonproliferative diabetic retinopathy, PDR: proliferative diabetic retinopathy, U: "unreadable" due to refractive media opacity.

The diagnostic results of the 6854 eyes in the ophthalmologists’ diagnosis group and the AI diagnosis group are shown in Table 3. As calculated from the data in Table 3, with respect to the ophthalmologists’ diagnosis of DR, the sensitivity and specificity of the AI diagnosis were 81.2% (95% confidence interval (CI): 80.3%-82.1%) and 94.3% (95% CI: 93.7%-94.8%), respectively, and the positive and negative predictive values were 80.4% (95% CI: 78.4%-82.4%) and 94.5% (95% CI: 93.9%-95.1%), respectively. The DR diagnostic screening results of the ophthalmologists and AI showed good consistency (κ = 0.752, *P*<0.05), and the DR diagnostic rate between the two methods were nearly identical, suggesting that the two methods have strong agreement and can be used in the same study in similar areas, thus supporting the application of AI software in DR screening. There was no significant difference in accuracy between the two methods (χ2 = 0.329, *P* = 0.566, *P*>0.05), suggesting that the two methods were complementary.

**Table 3:** Comparison of the diagnoses made by the ophthalmologists and AI (eye-level).

Ophthalmologists’ diagnosis	AI diagnosis system		Total
	DR	No DR	
DR	1254	305	1559
No DR	290	5005	5295
Total	1544	5310	6854

Note: κ = 0.752, DR: diabetic retinopathy (κ consistency check).

Considering that we are attempting to promote early screening, we performed a person-level analysis to compare the ophthalmologists’ and AI results, using the less severely diseased eye for the final diagnosis (Table 4). The sensitivity and specificity of the AI system were 84.6% (95% CI: 81.1–87.6%) and 95.0% (95% CI: 94.2%-95.7%), respectively, and its positive and negative predictive values were 72.2% (95% CI: 68.3%-75.7%) and 97.6% (95% CI: 97.0%-98.1%), respectively. The DR diagnostic screening results of the ophthalmologists and AI showed good consistency (κ = 0.742, *P*<0.05). Then, considering the correlation between the two eyes, we performed another person-level analysis, randomly selecting the diagnosis of one eye as the final diagnosis (Table 5). The sensitivity and specificity of the AI were 85.3% (95% CI: 82.8–87.5%) and 93.7% (95% CI: 92.8%-94.6%), respectively, and the positive and negative predictive values were 81.4% (95% CI: 78.8%-83.8%) and 95.2% (95% CI: 94.3%-95.9%), respectively. As with the previous person-level analysis, the DR diagnostic screening results of the ophthalmologists and AI showed good consistency (κ = 0.777, *P*<0.05). In addition, 96 of the 577 excluded eyes were identified and diagnosed by the ophthalmologists. Including these eyes in the analysis, random eyes were counted for the final result by individual. For the convenience of calculation, the results of the contralateral eyes were taken as the final result for the eyes that could not be diagnosed by AI; the sensitivity and specificity were 81.2% (95% CI: 79.2%-83.1%) and 93.8% (95% CI: 93.1%-94.4%), respectively. Although the specificity was reduced, the ophthalmologists and AI were still considered to have a good consistency.

**Table 4:** Comparison of the diagnoses made by the ophthalmologists and AI (person-level).

Ophthalmologists’ diagnosis	AI system diagnosis		Total
	DR	No DR	
DR	428	165	593
No DR	78	3125	3203
Total	506	3290	3796

Note: κ = 0.742, DR: diabetic retinopathy (κ consistency check).

**Table 5:** Comparison of the diagnoses made by the ophthalmologists and AI (person-level: Randomly selected eyes).

Ophthalmologists’ diagnosis	AI system diagnosis		Total
	DR	No DR	
DR	787	180	967
No DR	136	2693	2829
Total	923	2873	3796

Note: κ = 0.777, DR: diabetic retinopathy (κ consistency check).

In clinical practice, at least moderate NPDR requires intervention, while mild DR only requires blood glucose control and regular follow-up. Therefore, we compared the diagnoses of moderate and severe NPDR and PDR requiring intervention with that of mild NPDR (which does not require intervention) (Table 6). A satisfactory consistency was achieved between the AI screening system and the ophthalmologists’ examinations (κ = 0.621, *P*<0.05), although the AI performed slightly worse.

**Table 6:** Comparison of clinical outcomes requiring intervention.

Ophthalmologists’ diagnosis	AI system diagnosis		Total
	No DR or mild NPDR	Moderate or severe NPDR	
No DR or mild NPDR	5606	111	5717
Moderate or severe NPDR	507	630	1137
Total	6113	741	6854

Note: κ = 0.621, DR: diabetic retinopathy, NPDR: nonproliferative diabetic retinopathy (κ consistency check).

**Fig 5:**
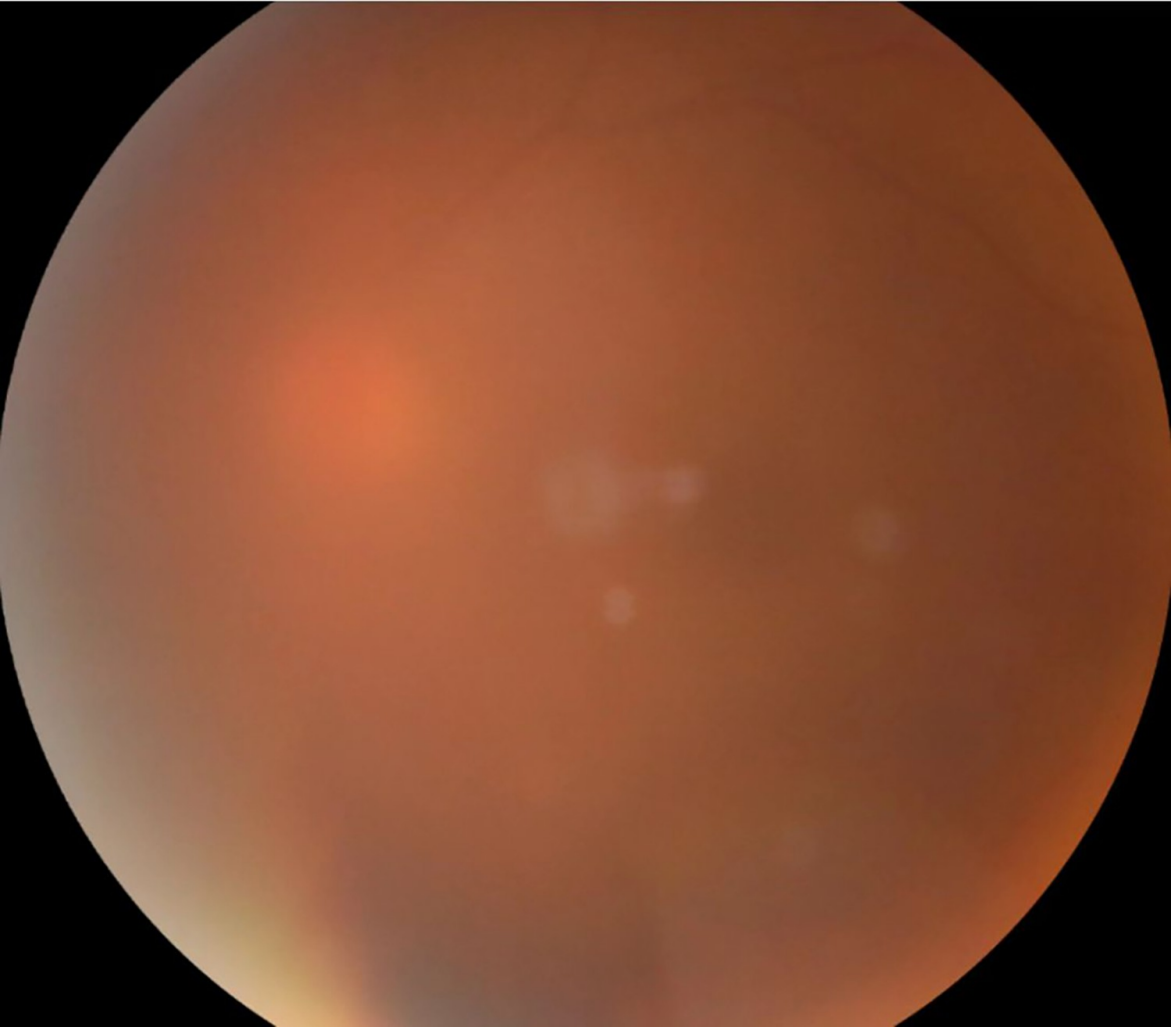
A case in which the AI misdiagnosed PDR as NDR.

Finally, we compared the performance for the two counties (Table 7), and the results showed moderate and good consistency for Lucheng (κ = 0.673, *P*<0.05) and Licheng (κ = 0.907, *P*<0.05), respectively. For Licheng, the sensitivity and specificity were 93.2% (95% CI: 90.5%-95.2%) and 98.0% (95% CI: 97.2%-98.5%), respectively. The sensitivity and specificity were 75.5% (95% CI: 72.7%-78.0%) and 92.0% (95% CI: 91.0%-92.9%), respectively, for Lucheng. We also examined the misdiagnosed eyes and the 577 eyes excluded by the AI. We found that image quality played a significant role in these cases; some images could be recognized by the AI but not accurately diagnosed due to refractive media opacity. Fig 5 shows an example of a case of PDR misdiagnosed as NDR by the AI system.

**Table 7:** Comparison of the prevalence of DR according to the different diagnostic methods in the two counties.

Ophthalmologists’ diagnosis	AI system diagnosis			
	Lucheng		Licheng	
	DR	No DR	DR	No DR
DR	788	264	466	41
No DR	256	3028	34	1977
Total	1044	3292	500	2018

Note: κ1 = 0.673, κ2 = 0.907, DR: diabetic retinopathy (κ consistency check).

## Discussion

This study assessed the accuracy of AI automatic analysis software in DR screening. To the best of our knowledge, large-scale DR screening in rural areas has not been conducted in central and western China, and the results of this study showed no obvious difference between the AI and ophthalmologists’ classification results. Therefore, AI-assisted DR screening can be feasibly applied and developed in rural hospitals.

Studies have shown that the prevalence of DR in eastern China and rural areas is higher than that in central and western China and cities, respectively, which may be related to income, education, medical conditions and dietary habits [3, 8, 21, 22]. However, the results of our study showed that the differences in age, sex and DR prevalence of nonurban residents living below the county level between the two counties were not significant, which may be because of similar dietary habits, medical conditions and small sample sizes in the two areas investigated. In our study, the prevalence of DR was 22.7%, which is different from that reported in the epidemiological study of Wang et al. [18] on diabetic retinopathy in the eastern rural area of Changzhi, Shanxi Province, which indicated that the prevalence of DR in Changzhi was 37.46%, possibly due to the increased awareness of fundus complications in DM patients. In contrast, in this study, DM patients whose DR grade could not be estimated due to fundus laser treatment and those with refractive media opacity due to cataracts, vitreous hemorrhage and other reasons were excluded, resulting in a low prevalence of DR. In addition, based on the findings of a meta-analysis on the prevalence of DR in thirty-one Chinese studies, the prevalence of DR in any DM patient was 18.45% [3]. In India, the prevalence of DR in type 2 DM patients was 17.6% [23], while in an American DM population, the incidence of any type of DR was 33.2% [24]. By summarizing the data from thirty-five population-based studies from around the world, the prevalence of any DR was found to be 34.6% [1]. The regional differences in DR may be related to research methods, geographical characteristics, demographic characteristics, and how people identify and classify DR.

The results of the DR Study Group and the Early Treatment DR Study Group confirmed that timely and effective treatment can prevent severe vision loss in 90% of DR patients and reduce the blindness rate from 50% to less than 5% [25]. Initial fundus results were obtained quickly due to the AI diagnosis system for DR screening, thus reducing the diagnosis and treatment time and providing timely referral to superior hospitals, making the system more efficient for ophthalmologists and patients. This new technology has aroused great interest in AI technology for DR screening worldwide [26]. In addition, we should note that our results are less satisfactory than the data obtained during model development. Our results suggested that the specificity was approximately 93%, which was comparable to that reported by the AI producer. However, our sensitivity was relatively low compared with that claimed by the producer (81.2% vs. 93%). This discrepancy might be due to the different types of fundus cameras used in the development process and screening in our study, and in part, to certain differences between the accuracy claimed by the camera developers and reality. With the rapid development of AI, an increasing number of AI software programs have been developed for DR screening. In 2013, Abràmoff et al. [13] published a DR screening program (IDP program) in Iowa, France, which had a sensitivity of 96.8%, a specificity of 59.4%, and an area under the curve of less than 0.937 in identifying timely referred diabetic retinopathy (referable diabetic retinopathy, RDR, defined as moderate NPDR or above or macular edema). In recent years, DL technology has emerged as a new branch of ML technology in the field of AI, capable of identifying complex structures in data without a predefined set of rules via multiple functions and the simultaneous processing of multidimensional data. One of these architectures is the CNN, which is inspired by the fact that the brain can learn complex data patterns through changes in the strength of synaptic connections between neurons [27]. DL involved training a neural network (a large function with millions of parameters) to perform a given task. The function calculates the severity of DR based on the intensity of pixels in the fundus image and then automatically grades the DR. However, building and training the software requires many fundus photos and millions of pathological changes, including knowledge of the severity of the DR, which is called the training set. In the process of training, the parameters of the neural network are originally set to random values, and then for each fundus photo, provided the function of the severity level compared with the known level, the parameters of the modified function are fine-tuned to reduce identification errors. After repeated training, the result is a sufficiently general function for calculating the level of DR for new images so that the AI diagnosis system can obtain a more accurate DR classification [8]. In 2016, the specificity of RDR detection was improved by using a CNN to screen DR [14]. The Zhiyuan Huitu fundus image AI analysis software used in this study uses the deep convolutional neural network Inception-V3 to perform automatic DR grading for fundus color images. This CNN uses a function to first combine adjacent pixels into partial features and then aggregates these features together to global features. Although the algorithm does not explicitly detect lesions (such as bleeding and microaneurysms), it may learn to identify them using local features, further improving the sensitivity and specificity for DR diagnosis. Using AI for DR screening not only overcomes the barriers of screening difficulties, improves screening efficiency and reduces the need for ophthalmologists for fundus disease but also has high accuracy and alleviates the screening needs for a large number of DM patients.

At present, there are many studies on the application of AI in DR screening. Based on fundus photography, Abràmoff et al. [14] showed a sensitivity of 96.8%, a specificity of 87%, and an AUC of 0.937 using fundus photos. Ting et al. [28] found that the sensitivity, specificity and AUC of AI diagnosis based on fundus photos were 90.5%, 91.1% and 0.936, respectively. Gulshan et al. [9] obtained a sensitivity of 97.5%, a specificity of 93.4%, and an AUC of 0.991. The study by Li et al. [16] showed that the consistency rate of the DR-screening AI system could reach the level of ophthalmologists with senior professional titles and greatly shorten the reading time, which could provide a reliable method and platform for large-scale DR screening for diabetic patients. Therefore, the application of AI in DR screening has high sensitivity and specificity, saves time and improves screening efficiency. In addition, all cloud-based software programs need high computing capacity, especially network connections, to achieve living reporting. The latest AI system for diagnosing DR is Medios, an offline AI algorithm based on smartphones from India [29]. That study confirmed that the AI-based software has high sensitivity and specificity in detecting DR. In this study, statistical analysis showed that the sensitivity and specificity of the AI system in detecting the presence of DR were 81.2% and 94.3%, respectively, indicating a low sensitivity and specificity, similar to the study of Ting et al. [28], which may be because their study was similar to ours and closer to clinical practice. In addition, in this study, the consistency rate between the AI diagnosis system and the ophthalmologists’ diagnosis was 81.6%, which basically meets the needs of hospitals in rural areas for DR screening. The ophthalmologists and AI-assisted DR screening methods were consistent, achieving κ values similar to the AI-assisted DR screening study conducted by Qin et al. [30] in primary hospitals, but our diagnostic consistency rate is higher. A recent AI algorithm from Taiwan [31] also showed higher sensitivity than physicians and ophthalmologists in diagnosing DR, but the ophthalmologists had higher specificity, which may be more meaningful for primary hospitals because they have more physicians than ophthalmologists. In this study, the diagnostic result was 0.752 (*P*<0.05), suggesting that the method of DR screening conducted by AI has strong repeatability and can be used for identical studies in similar areas, thus promoting the application of AI software in DR screening to the benefit of more rural areas. More DM patients can be screened by DR to achieve early prevention, early diagnosis and early treatment. However, according to this study, there was a discrepancy in the AI-ophthalmologist consistency between Lucheng and Licheng (Kappa value, 0.673 vs. 0.907). The possible reason for this discrepancy might be that the population bases of the two counties were different and the prevalence of DR was also different. Nevertheless, our study showed that the overall consistency rate between AI and ophthalmologists was satisfactory (81.6%), and therefore, this AI system can be used for DR screening in rural areas.

This study also had some limitations. New DM patients who had not been included in the National Basic Public Health Information System of Changzhi were not included in this study. Although the AI software can automatically grade retinal images, it cannot overcome physiological limitations, such as unsatisfactory retinal photos from patients with small pupils and cloudy lenses. Second, the AI-based diagnostic system has difficulty diagnosing macular cystoid edema with only fundus color images. Therefore, the AI-based diagnostic system cannot accurately classify macular cystoid edema caused by DR; this matter is an important drawback that will be improved in the future.

In conclusion, compared with that of the ophthalmologists’, the sensitivity of the AI software system in screening DR was slightly lower, but the difference in the diagnostic accuracy rate was not obvious; additionally, the AI system had a similar specificity to that achieved in other research studies. The AI diagnosis system for screening DR in rural areas is feasible, but the accuracy needs to be further improved. Establishment of the AI system requires the reading of and training with many images, but this will help alleviate ophthalmologists’ imaging reading habits and will be conducive to DR patients in rural areas receiving timely, effective and accurate treatment. The AI software in this study needs to be further improved to improve its the photo reading quality and accuracy to provide a method and platform for large-scale DR screening.

## Supporting information

S1 File (XLSX)

S2 File (DOCX)

S3 File (DOCX)
