# Supplementary material for: Clinical evaluation of AI-assisted screening for diabetic retinopathy in rural areas of midwest China
Source: PLoS One. 2022 Oct 13;17(10):e0275983. doi: 10.1371/journal.pone.0275983 (PMC9560484; doi:10.1371/journal.pone.0275983)
Supplement: S2 File — (DOCX) [file pone.0275983.s002.docx]

**基于人工智能诊断系统的糖尿病视网膜病变筛查模式研究-研究计划**

**研究背景：**

糖尿病视网膜病变（DR）是糖尿病（DM）的主要并发症；这是全世界工作年龄段视力丧失的主要原因。全世界糖尿病患者中DR的患病率为34.6%，其中10.2%患有视力受损的视网膜病变[1]。在中国，糖尿病的患病率为11.2%，约23%的糖尿病患者发生DR[2]；此外，在DR人群中，非增殖性DR占19.1%，威胁视力的增殖型DR占2.8%[3]。就发病年龄而言，DR的发病率在60至69岁之间达到高峰；它还随着原发性疾病的病程而增加[4]。由于DR会导致失明，因此在DM患者中早期筛查DR，然后及时治疗，以延缓疾病进展，从而降低DR导致的失明率，具有重要的临床意义。然而，传统的人工DR筛查需要训练有素的辅助医务人员和大量初级卫生工作者。此外，卫生当局的关注可能构成DR筛查的另一个阻碍因素。因此，寻找一种有效的方法进行广泛的DR筛查具有重要的临床和社会意义。

人工智能最早提出于20世纪50年代。随着数据量的指数增长和计算机数据处理能力的提高，近年来人们对人工智能的兴趣重新燃起。人工智能于20世纪70年代首次引入医学领域；从那时起，许多人工智能系统不断涌现[6]人工智能也被应用于眼科领域[7]。2016年，Google Deep Mind开发了一个机器学习系统，可以自动识别DR患者的眼底彩色照片。AI诊断系统在DR筛查中的应用大大提高了筛查效率和医疗资源的利用率，从而减轻了社会、医疗系统和患者的负担[8]。

目前，基于人工智能的DR筛查已在法国、英国、日本、辛家坡和新西兰等发达国家以及印度等发展中国家开展[9-14]。然而，这些工作大多是国家计划的一部分，例如，美国爱荷华州的爱荷华州探测计划（IDP）[15]。虽然有一些关于中国DR筛查的研究报道，但大多数是在大城市的社区医院进行的；在缺乏必要眼科设备的农村地区，尚未进行大规模筛查[16，17]。

**为什么中国中西部的农村地区需要这项研究？**

中国中西部地区显示出从早期DR筛查和干预中获得重大健康收益的巨大潜力，原因如下。

（1） 2019年，华东地区人均GDP为9.64万元，中西部地区人均GDP分别为4.87万元和5.41万元；也就是说，与华东地区相比，中西部地区的经济水平相对较低。因此，中国中西部的大多数人负担不起DR治疗和终身随访。此外，在中国，农村地区的糖尿病总患病率高于城市地区[1]，约87%的糖尿病患者在县级以下医院接受治疗；

（2） 医疗领域的资源分布不均，与眼科相关的资源相对匮乏。传统上，在中国中西部农村地区，DR筛查和诊断必须求助于眼科医生。然而，在这些地区，眼科医生的短缺问题十分突出：中国东部、中部和西部县级以下医疗机构的每千人口眼科医生人数分别为0.02、0.012和0.011。在被调查城市所属的山西省，该比率为0.035；

（3） 大多数糖尿病患者为中年或老年人。与中国东部经济发达地区相比，中国中西部地区人群对糖尿病视网膜病变的认识和预防相对薄弱。因此，对于中国中西部人群，基于DR筛查结果的健康教育和干预将有助于提高患者对DM相关眼底并发症的认识；

（4） 人工DR筛查工作量大，人工智能技术已接近成熟。Abramoff等人在2016年进行的一项研究表明，用于DR筛查的AI系统实现了令人满意的敏感性和特异性（分别为97%和87%）[9]。此外，根据Tommy等人的研究，人工智能单页阅读的平均时间可短至1.62±0.67秒，这与具有高级职称的经验丰富的眼科医生所花费的时间相当[18]。因此，使用人工智能进行DR筛查可以大大减少纸张阅读时间，提高工作效率；

（5） 医疗资源在我国东、中、西部地区分布不均。然而，在中国中西部，几乎每个村庄都可以访问互联网，这是一个优势，可以为进行DR筛查提供有利条件；和

（6） 长治是中国中西部的一个典型城市。对该市农村地区的糖尿病患者进行DR筛查将有助于大量无法及时接受治疗的糖尿病患者。这样的筛查也将有助于长治市大型DR健康数据库的建设，为政府制定DR健康相关政策提供参考。此外，本研究所建立的DR筛查模式不仅对中国中西部其他农村地区，而且对其他具有类似社会经济背景的国家的地区都具有很大的应用价值。

**方法/设计**

***研究假设***

1、基于人工智能的系统的建立将有利于大范围早期DR筛查的深入，降低DR的致盲率和致残率；

2、在中国中西部农村地区建立基于人工智能的DR筛查模式，将提供及时的DR检测手段，从而降低无法承担DR终身随访和治疗费用的患者的成本。

3、基于人工智能的DR筛查系统的建立将提高中国中西部人群对DR预防的认识。

***目的和目标***

本研究的目的是在长治市农村地区建立一种基于人工智能诊断系统的DR筛查模式，并最终将该模式推广到中国中西部其他农村地区。具体目标如下：

1、建立适合长治市农村的DR筛查模式，有利于进一步建立DR综合防治体系；

2、在涉及地区的已知糖尿病登记人群中完成大规模筛查。

3、建立调查地区大型DR健康相关数据库，为政府制定相关卫生政策提供参考。

4、在中国中西部地区以及与本研究调查地区社会经济特征相似的其他国家地区的DR筛查实践中推广筛查模式。

***研究设计***

这项前瞻性队列研究计划于2021 7月1日开始，2021年12月结束。截至2021 7月1日，已在长治市国家基本公共卫生信息系统登记的糖尿病患者被纳入目标人群。在研究开始时，共有79117名糖尿病患者已在城市系统中登记。长治市辖11个县。在这些县中，分别代表长治市经济水平较高和经济水平较低的两个县——屯留县和黎城县被选中，来自这两个县的注册糖尿病患者构成筛查组。屯留和历城登记的糖尿病患者人数分别为5377和3353。

本研究使用的致远汇图眼底图像AI（识别号：CN/BJS 235635）分析软件是欧盟认证的第一批眼科AI医疗产品，获得国际市场认可。所有纳入患者的联系信息通过长治市基本公共卫生信息系统获取，糖尿病患者由负责患者所在社区（村）的全科医生联系。DR筛查在指定时间在当地社区医院进行。对于每位患者，拍摄两张以黄斑和视盘为中心的45度照片，然后将其上传到AI系统中，以便根据国际DR分级标准（2002年版）进行基于DR的筛查和分级。对于诊断为DR的患者，将根据病情的严重程度提供适当的干预。

***纳入/排除标准***

纳入标准如下：1）受试者必须了解临床试验的目的并自愿参与；签署知情同意书（对于老年患者，可获得其监护人的同意）；2） 1型糖尿病和2型糖尿病患者的性别和年龄≥ 18年。

符合以下任何标准的患者将被排除在本研究之外：1）黄斑水肿；2） 因严重身体和/或其他疾病无法配合检查；3） 根据眼底检查，屈光性中膜混浊，如角膜溃疡、角膜白斑、严重白内障、玻璃体出血和大量渗出；4）采集图像质量差。

***临床决策***

基于DR大数据智能诊断平台的DR筛查模式决策分析包括健康管理决策分析和临床决策分析。健康管理决策分析旨在根据DR的识别率、患者的筛查依从性和支付意愿，评估筛查模式的可行性和有效性。根据AI诊断系统的筛查和诊断结果，进行临床决策分析，为DR的精确分级干预提供依据，并形成DR治疗的临床决策。

***数据分析***

将使用SPSS 22.0统计软件对数据进行分析。灵敏度、特异性和曲线下面积（AUC）将用于预测和评估AI诊断系统的性能。平均值和标准偏差将用于数值变量。对于顺序变量，将使用卡方检验进行差异检验；对于正态分布的数值变量，将使用t检验或ANOVA进行差异检验。其他非参数分析将酌情使用Mann-Whitney U检验、Wilcoxon符号秩检验和Wilcoxon秩和检验。将使用逻辑回归方法进行多变量分析。

**研究意义**

虽然一些国家已经开展了基于人工智能诊断系统的DR筛查，但大多数做法都是国家计划的一部分，或者是在大城市的社区医院开展的。据我们所知，尚未在农村地区进行大规模DR筛查。

本研究以长治市农村地区为目标地区，主要目的是为中国中西部农村地区提供一种新的基于人工智能的DR筛查模式。这种模式也适用于非眼科专业培训，这将有助于开展DR筛查，以及眼科医疗资源短缺地区的DM患者。根据这一大规模筛查的结果，可以规划及时的治疗，这可以节省治疗费用，从而减轻社会负担。此外，虽然这项研究是在中国进行的，但它可以为具有类似医疗和社会经济背景的国家和地区提供参考。

**参考文献：**

1. Yau JWY, Rogers SL, Kawasaki R, et al. Global Prevalence and Major Risk Factors of Diabetic Retinopathy. Diabetes Care 2012; 35(3): 556-564.

2. Li Y, Teng D, Shi X, et al. Prevalence of diabetes recorded in mainland China using 2018 diagnostic criteria from the American Diabetes Association: national cross sectional study. Bmj 2020; 369: m997.

3. Xu Y, Wang L, He J, et al. Prevalence and control of diabetes in Chinese adults. Jama 2013; 310(9): 948-959.

4. Ebneter A, Zinkernagel MS. Novelties in Diabetic Retinopathy. Endocr Dev 2016; 31: 84-96.

5. Broome DT, Hilton CB, Mehta N. Policy Implications of Artificial Intelligence and Machine Learning in Diabetes Management. Curr Diab Rep 2020; 20(2): 5.

6. Gulshan V, Peng L, Coram M, et al. Development and Validation of a Deep Learning Algorithm for Detection of Diabetic Retinopathy in Retinal Fundus Photographs. Jama 2016; 316(22): 2402-2410.

7. Ting DSW, Pasquale LR, Peng L, et al. Artificial intelligence and deep learning in ophthalmology. Br J Ophthalmol 2019; 103(2): 167-175.

8. Brown AF, Jiang L, Fong DS, et al. Need for eye care among older adults with diabetes mellitus in fee-for-service and managed Medicare. Arch Ophthalmol 2005; 123(5): 669-675.

9. Abràmoff MD, Lou Y, Erginay A, et al. Improved Automated Detection of Diabetic Retinopathy on a Publicly Available Dataset Through Integration of Deep Learning. Invest Ophthalmol Vis Sci 2016; 57(13): 5200-5206.

10. Raman R, Srinivasan S, Virmani S, et al. Fundus photograph-based deep learning algorithms in detecting diabetic retinopathy. Eye (Lond) 2019; 33(1): 97-109.

11. Takahashi H, Tampo H, Arai Y, et al. Applying artificial intelligence to disease staging: Deep learning for improved staging of diabetic retinopathy. PLoS One 2017; 12(6): e0179790. 12. Quellec G, Charrière K, Boudi Y, et al. Deep image mining for diabetic retinopathy screening. Med Image Anal 2017; 39: 178-193.

13. Ting DSW, Cheung CY, Lim G, et al. Development and Validation of a Deep Learning System for Diabetic Retinopathy and Related Eye Diseases Using Retinal Images From Multiethnic Populations With Diabetes. Jama 2017; 318(22): 2211-2223.

14. Rajalakshmi R, Subashini R, Anjana RM, et al. Automated diabetic retinopathy detection in smartphone-based fundus photography using artificial intelligence. Eye (Lond) 2018; 32(6): 1138-1144.

15. Abràmoff MD, Folk JC, Han DP, et al. Automated analysis of retinal images for detection of referable diabetic retinopathy. JAMA Ophthalmol 2013; 131(3): 351-357.

16. He J, Cao T, Xu F, et al. Artificial intelligence-based screening for diabetic retinopathy at community hospital. Eye (Lond) 2020; 34(3): 572-576.

17. Wang XN, Dai L, Li ST, et al. Automatic Grading System for Diabetic Retinopathy Diagnosis Using Deep Learning Artificial Intelligence Software. Curr Eye Res 2020: 1-6.
