# Supplementary material for: Clinical evaluation of AI-assisted screening for diabetic retinopathy in rural areas of midwest China
Source: PLoS One. 2022 Oct 13;17(10):e0275983. doi: 10.1371/journal.pone.0275983 (PMC9560484; doi:10.1371/journal.pone.0275983)
Supplement: S3 File — (DOCX) [file pone.0275983.s003.docx]

**Research on screening mode of diabetes retinopathy based on artificial intelligence diagnosis system - research protocol**

**Background**

Diabetic retinopathy (DR) is a major complication of diabetes mellitus (DM); it is a main cause of loss of vision at work age across the world. The prevalence of DR among DM patients is 34.6% worldwide, among whom 10.2% suffer visually impaired retinopathy [1]. In China, the prevalence of DM is 11.2%, and approximately 23% of DM patients develop DR [2]; in addition, of the DR population, non-proliferative DR accounts for 19.1% and the vision-threatening proliferative type accounts for 2.8% [3]. In terms of age of onset, the incidence of DR peaks between 60 years and 69 years; it also increases with the course of the primary disease [4]. As DR can lead to blindness, early screening of DR among DM patients, following which timely treatment can then be provided to delay disease progression, thereby reducing the DR-caused blindness rate, is of great clinical significance. However, traditional artificial DR screening requires well-trained paramedical staff and a large number of primary level health workers. In addition, attention from health authorities may constitute another impeding factor for DR screening. Therefore, finding an effective way to conduct wide-range DR screening is of great clinical and social significance.

AI was first proposed in the 1950s. With an exponential increase in data amounts and the improvement of data-processing capacity of computer, people's interest in AI has been rekindled in recent years [5]. AI was first introduced into the medical field in the 1970s; since then, a number of AI systems have been emerging [6] AI has also been applied in the field of ophthalmology [7]. In 2016, Google Deep Mind developed a machine learning system, which can automatically recognizes the fundus color photos of DR patients. The application of AI diagnostic system in DR screening greatly enhances screening efficiency and the utilization rate of medical resources thereby reducing the burdens of the society, medical system and patients [8].

Currently, AI-based DR screening has been carried out in developed countries such as France, Britain, Japan, Xinjiapo and New Zealand, as well as in developing countries such as India [9-14]. However, most of these works are a part of national plans, e.g., the Iowa Detection Program (IDP) in Iowa, US [15]. Although a few studiesabout DR screening in China have been reported, most were conducted in community hospitals in large cities; large-scale screening in rural areas, where necessary ophthalmic equipment lacks, has not been conducted [16, 17].

**Why is this study needed in rural areas of Midwest China?**

Midwest China exhibits a huge potential to achieve major health gains from early DR screening and intervention, and the reasons are as follows.

(1) In 2019, the per capita GDP of eastern China was 96,400 yuan (RMB), whereas those of central and western China were 48,700 yuan and 54,100 yuan, respectively; that is, the economic levels of central and western China were relatively low, compared with that of eastern China. For this reason, most people in Midwest China cannot afford DR treatment and life-long follow-ups. In addition, in China, the overall prevalence rate of DM in rural areas is higher than that in urban areas [1], and approximately 87% DM patients receive treatment in hospitals below the county level;

(2) Resource distribution is uneven across medical fields, and the resources related to ophthalmology are relatively deficient. Traditionally, DR screening and diagnosis have to resort to ophthalmologists in the rural areas of Midwest China. However, in these areas, the shortage of ophthalmologists is prominent: The numbers of ophthalmologists per thousand population in the medical institutions below the county level in eastern, central and western China are 0.02, 0.012, and 0.011, respectively. In Shanxi Province, which the investigated cities belong to, the ratio is 0.035;

(3) Most DM patients are middle-aged or older. Compared with the economically developed regions in the eastern China, the awareness and prevention of diabetic retinopathy among the populations in the regions of Midwest China are relatively weak. Therefore, for the populations in Midwest China, health education and intervention based on DR screening outcomes will be helpful to increase patients’ awareness of DM-associated fundus complications;

(4) The workload of manual DR screening is heavy, and AI technology has approached to maturation. A study conducted by Abramoff et al. in 2016 showed that AI system for DR screening achieved satisfactory sensitivity and specificity (97% and 87%, respectively) [9]. In addition, according to Tommy et al, the average time of single-sheet reading by AI can be as short as 1.62±0.67 s, which is comparable to the time consumed by experienced ophthalmologists with a senior title [18]. Therefore, the use of AI for DR screening can greatly reduce sheet-reading time and improves work efficiency;

(5) Medical resources are not distributed evenly among the eastern, central and western regions of China. Nevertheless, in Midwest China, the Internet can be accessed almost in each village, which is an advantage that can provide favorable conditions for the to-be-conducted DR screening; and

(6) Changzhi is a typical city located in Midwest China. DR screening among the DM patients in the rural areas of this city will be useful for a substantial number of DM patients who cannot receive timely treatment. Such a screening will also be helpful for the construction of a large DR-health database of Changzhi City, which may provide reference for the government to enact DR health-related policies. In addition, the established DR screening mode in this study may be of great application value not only for other rural areas of Midwest China but also for the regions of other countries with similar socioeconomic backgrounds.

**Addressing system barriers**

In the central and western regions of China, health services are less developed, compared with those in eastern China. There, healthcare system refers primarily to hospital-based care and treatment for the individuals who can afford treatment. In addition, such a system often target DM, and its contribution to comprehensive prevention and treatment of the complications of DM, such as DR, is small.

Only 40%-45% of the whole DM population can receive early detection and diagnosis of DM [19]. Additionally, the onset of DR is insidious with few apparent symptoms. For most DM patients that come to hospital for fundus lesion examination, DR has already progressed to a moderate or even severe stage [20].

In the mode proposed in this study, the AI diagnostic system will be utilized for DR screening. According to this mode, non-ophthalmology professional medical staff in primary hospitals can carry out DR screening and intervention, such as s endocrine therapy and intravitreal medication, which will reduce the harm of DR.

**Methods/Design**

**Research hypothesis**

1. The establishment of the AI-based system will benefit the depth of the wide-range early DR screening, and the blindness and disability rates of DR can be reduced;

2. The establishment of an AI-based mode for DR screening in the rural areas of Midwest China will provide a timely DR detection means, and therefore reduce costs for patients who cannot afford lifelong follow-ups and treatment of DR.

3. The establishment of the AI-based DR screening system will enhance the awareness of the populations in Midwest China about DR prevention.

**Aim and objectives**

The aim of our research is to establish a DR screening mode in the rural areas of Changzhi City based on an AI diagnosis system, with the final expectation to extend this mode to other rural areas in Midwest China. The specific objectives are as follows:

1. To establish a DR screening mode suitable for the rural areas of Changzhi City, which will be beneficial for further establishment of a comprehensive prevention and treatment system of DR;

2. To complete large-scale screening in registered known diabetic population in the involved areas.

3. To establish a large DR health-related database of the investigated region, which may provide reference for the government to formulate related health policies.

4. To promote the screening mode in the practice of DR screening in the central and western regions of China, as well as in the regions of other countries with socioeconomic characteristics similar to the investigated regions in this study.

**Study design**

This prospective cohort study is planned to begin on July 1, 2021 and end in December 2021. The DM patients that have already registered in the National Basic Public Health Information System of Changzhi City by July 1, 2021, are included as the target population. At the beginning of the study, a total of 79,117 DM patients have already been registered in the system of the city. Changzhi City contains 11 counties. Among these counties, Tunliu and Licheng that represent the counties at a relatively high economic level and a low economic level, respectively, in Changzhi City are selected, and the registered DM patients from these two counties constitute the screening group. The numbers of the registered DM patients from Tunliu and Licheng are 5,377 and 3,353, respectively.

The Zhiyuan Huitu fundus image AI (identification number: CN/BJS 235635) analysis software used in this study is a first-batch ophthalmic AI medical product certified by the European Union and recognized by the international market. The contact information of all the included patients is obtained through the basic public health information system of Changzhi City, and the DM patients are contacted by the general practitioner who is in charge of the community (village) where the patients live. DR screening is performed at the local community hospital at an appointed time. For each patient, two 45-degree photos centered on the macula and on the optic disc are taken, which are then uploaded into the AI system for DR-based screening and grading according to the international grading criteria for DR (version, 2002). For patients diagnosed with DR, appropriate intervention will be provided according to the severity of the condition.

**Inclusion/exclusion criteria**

The inclusion criteria are as follows: 1) The subject must be aware of the purpose of the clinical trial and participate voluntarily; he/she signs the informed consent form (for elderly patients, consent may be obtained from their guardians); 2) Patients with type 1 DM and type 2 DM of either sex with age ≥ 18 years.

Those meeting any of the following criteria will be excluded from this study: 1) macular edema; 2) unable to cooperate with the examination due to serious physical and/or other diseases; 3) refractive media opacity, such as corneal ulcers, corneal leukoplakia, severe cataracts, vitreous hemorrhage and massive exudation, according to fundus examination; and 4) poor quality of the acquired image.

**Clinical decision-making**

Decision analysis of the DR screening mode based on the DR big data intelligent diagnosis platform includes health management decision analysis and clinical decision analysis. Health management decision analysis aims to evaluate the feasibility and effectiveness of the screening mode based on the recognition rate of DR and patients’ screening compliance and willingness to pay. Based on the screening and diagnostic outcomes of the AI diagnosis system, clinical decision analysis is performed to provide a basis for precise grading intervention on DR and to form clinical decision-making for DR treatment.

**Quality control**

1. Technical training quality control: Paper test and practice assessment will be performed after training, and screening can only be carried out after the trained staff passes the examinations.

2. Screening quality control: To control image quality, 10% of the data are sampled for AI-based accuracy evaluation by ophthalmologists. For images in poor quality or difficult to grade, artificial reviews will be performed.

3. Baseline data of the population: Screening data are timely sorted, and due attention is also given to missing data.

4. Expert evaluation: Experts in the fields of epidemiology and health economics are invited to assess the collated cost data and give suggestions. Accordingly, further investigation, verification, and revisions may be performed.

**Data analysis**

SPSS 22.0 statistical software will be used to analyze the data. The sensitivity, specificity and area under curve (AUC) will be used to predict and evaluate the performance of the AI diagnosis system. The mean and standard deviation will be used for numerical variables. Differences will be tested using the Chi-square test for ordinal variables, and the *t* test or ANOVA for numerical variables with a normal distribution. Additional nonparametric analyses will be performed using the Mann-Whitney U test, the Wilcoxon sign-rank test and the Wilcoxon rank-sum test, as appropriate. Multivariable analyses will be performed using the logistic regression method.

**Significance**

Although AI diagnostic system-based DR screening has been carried out in some countries, most of the practices served as a part of national plans or were carried out in community hospitals of large cities. To the best of our knowledge, large-scale DR screening in rural areas has not been conducted.

In this study, the rural areas of the Changzhi city are selected as the target areas, with the primary aim to provide a new AI-based DR screening mode for the rural areas in Midwest China. This mode may also be useful for non-ophthalmology professional training, which will benefit the conduction of DR screening, as well as DM patients in areas where there is a shortage of ophthalmic medical resources. Based on the results of this large-scale screening, timely treatment can be planned, which may save treatment costs and thus reduces social burdens. In addition, although this study is carried out in China, it may provide a reference for countries and regions with similar medical and socio-economic backgrounds.

**References**

1. Yau JWY, Rogers SL, Kawasaki R, et al. Global Prevalence and Major Risk Factors of Diabetic Retinopathy. Diabetes Care 2012; 35(3): 556-564.

2. Li Y, Teng D, Shi X, et al. Prevalence of diabetes recorded in mainland China using 2018 diagnostic criteria from the American Diabetes Association: national cross sectional study. Bmj 2020; 369: m997.

3. Xu Y, Wang L, He J, et al. Prevalence and control of diabetes in Chinese adults. Jama 2013; 310(9): 948-959.

4. Ebneter A, Zinkernagel MS. Novelties in Diabetic Retinopathy. Endocr Dev 2016; 31: 84-96.

5. Broome DT, Hilton CB, Mehta N. Policy Implications of Artificial Intelligence and Machine Learning in Diabetes Management. Curr Diab Rep 2020; 20(2): 5.

6. Gulshan V, Peng L, Coram M, et al. Development and Validation of a Deep Learning Algorithm for Detection of Diabetic Retinopathy in Retinal Fundus Photographs. Jama 2016; 316(22): 2402-2410.

7. Ting DSW, Pasquale LR, Peng L, et al. Artificial intelligence and deep learning in ophthalmology. Br J Ophthalmol 2019; 103(2): 167-175.

8. Brown AF, Jiang L, Fong DS, et al. Need for eye care among older adults with diabetes mellitus in fee-for-service and managed Medicare. Arch Ophthalmol 2005; 123(5): 669-675.

9. Abràmoff MD, Lou Y, Erginay A, et al. Improved Automated Detection of Diabetic Retinopathy on a Publicly Available Dataset Through Integration of Deep Learning. Invest Ophthalmol Vis Sci 2016; 57(13): 5200-5206.

10. Raman R, Srinivasan S, Virmani S, et al. Fundus photograph-based deep learning algorithms in detecting diabetic retinopathy. Eye (Lond) 2019; 33(1): 97-109.

11. Takahashi H, Tampo H, Arai Y, et al. Applying artificial intelligence to disease staging: Deep learning for improved staging of diabetic retinopathy. PLoS One 2017; 12(6): e0179790. 12. Quellec G, Charrière K, Boudi Y, et al. Deep image mining for diabetic retinopathy screening. Med Image Anal 2017; 39: 178-193.

13. Ting DSW, Cheung CY, Lim G, et al. Development and Validation of a Deep Learning System for Diabetic Retinopathy and Related Eye Diseases Using Retinal Images From Multiethnic Populations With Diabetes. Jama 2017; 318(22): 2211-2223.

14. Rajalakshmi R, Subashini R, Anjana RM, et al. Automated diabetic retinopathy detection in smartphone-based fundus photography using artificial intelligence. Eye (Lond) 2018; 32(6): 1138-1144.

15. Abràmoff MD, Folk JC, Han DP, et al. Automated analysis of retinal images for detection of referable diabetic retinopathy. JAMA Ophthalmol 2013; 131(3): 351-357.

16. He J, Cao T, Xu F, et al. Artificial intelligence-based screening for diabetic retinopathy at community hospital. Eye (Lond) 2020; 34(3): 572-576.

17. Wang XN, Dai L, Li ST, et al. Automatic Grading System for Diabetic Retinopathy Diagnosis Using Deep Learning Artificial Intelligence Software. Curr Eye Res 2020: 1-6.
